# The complete mitochondrial genome of the Chinese mountain keelback (Natricidae: *Opisthotropis kuatunensis*), comparative and phylogenetic analysis

**DOI:** 10.1080/23802359.2021.1964397

**Published:** 2021-11-10

**Authors:** Wei Liu, Bin Cai, Qiu-Ran Xia, Yan-Qing Wu

**Affiliations:** a Nanjing Institute of Environmental Sciences, Ministry of Ecology and Environment, Jiangsu, Nanjing, China; b Scientific Monitoring Center, Administration Bureau of National Park of Wuyi Mountain, Fujian, Wuyishan, China; c School of Interdisciplinary Studies, University of Glasgow, Dumfries, Scotland, United Kingdom

**Keywords:** Mitochondrial genome, *Opisthotropis kuatunensis*, Phylogenetic analysis

## Abstract

*Opisthotropis kuatunensis* is classified in the family Natricidae and is widespread in southern China. In this study, we sequenced and analyzed the circular mitochondrial genome of *O. kuatunensis* from the Fujian Province, China. The complete mitogenome is 17,279 bp in length, and includes 13 protein-coding genes, 2 ribosomal RNAs, 22 transfer RNA, 1 non-coding region of an L-strand replication origin and 2 control regions (D-loop1 and D-loop2). Phylogenetic analysis based on the complete mitochondrial genome supported *Opisthotropis* as monophyletic and sister to Nerodia and fully resolved *O. kuatunensis* on a branch with *O. latouchii*. This study contributes to the systematics, phylogeny and taxonomy of the Natricidae.

*Opisthotropis kuatunensis* (Pope 1928) is distributed in southern China and dependent on inland waters, forests and wetlands (Wake et al. 1995). Due to the habitat loss from rapid urbanization and climate change, this species is under enormous threat. Though it is currently listed as a species of least concern by the International Union for Conservation of Nature, as an ecological indicator, this species deserves more protection (China Snakes Working Group 2014). Nowadays, only two complete mitochondrial genomes of *Opisthotropis* species are published (Wang et al. 2019; Zhang et al. 2019). The genetic diversity of this species is still unclear in the Natricidae due to limited genetic studies (Wang et al. 2017). In this study, we sequenced the mitogenome of *O. kuatunensis* using next-generation sequencing to determine its mitogenome structure and systematic relationship to other Natricidae.

The adult specimen of *O. kuatunensis* was gathered from Yanzhang Village (28.19217440°N, 118.99537°E), Longquan City, Fujian Province, China. The sample (Specimen voucher: LSU20200820LQGDHLS01) was deposited in the Museum of Laboratory of Amphibian Diversity Investigation of Lishui University (https://mail.163.com/, for more information about this voucher please contact Wei Liu, email:Lw_ecology@163.com). The genomic DNA was extracted from muscle tissue using the EasyPure genomic DNA kit (TransGen Biotech Co, Beijing, China). The raw sequence data (15.65 G) was deposited in NCBI’s Sequence Read Archive (SRA; accession: SRR13516389), and the whole genome sequencing (15.62 G) was carried out on the Illumina NovaSeq6000 platform (Novogene Bioinformatics Technology Co. Ltd., Tianjin, China) using paired-end 150 bp methods. The mitogenome was assembled using the NOVO Plasty 3.7 (Dierckxsens et al. 2017) and annotated by MITOS Web Server (Matthias et al. 2013). The assembled mitogenome was uploaded and is available in NCBI (GeneBank accession number: MW788642).

The complete mitochondrial genome of *O. kuatunensis* is a circular molecule 17,279 bp in length and, contains 13 protein-coding genes (PCGs), 2 ribosomal RNAs, 22 transfer RNAs, 1 non-coding region of an L-strand replication origin and 2 control regions (D-loop1 and D-loop2). The overall nucleotide composition is 35.04% of A, 25.63% of T, 12.48% of G and 26.86% of C. All protein-coding genes in the mitogenome use ATG start codons except for NDA (ATA), ND2 (ATT), ND3 (ATT), and COX1 (GTG). For 13 PCGs, four of these PCGs terminated with TAA, COX1 with AGG, ND2 and ND4 with TAG, ND6 with AGA, and five with incomplete stop codons (T-/T–), forming TAA by post-transcription polyadenylation (Anderson et al. 1981). The size, content and organization of the *O. kuatunensis* mitogenome is similar to other published genomes of Natricidae (Xu et al. 2016).

Phylogenetic analysis based on complete mitochondrial genome sequences was conducted, using nucleotide substitution model GTT + I + G and default settings in MrBayes v3.2. Three species, *Naja atra*, *Bungarus fasciatus* and *Pseudoxenodon stejnegeri*, were designated as outgroups. The Bayesian analysis indicated that *O. kuatunensis* is closely related to *O. latouchii*, *O. andersonii* and *Nerodia sipedon* (Figure 1). These data show that *Opisthotropis* and *Nerodia* may share the same ancestor. The p-distances between *O. kuatunensis* and other *Opisthotropis* species were both more than 7% based on the 13 PCGs via MEGA 5.05. This mitogenome obtained in this study enriches the genomic resources available and fully unravel the phylogenetic relationship among *Opisthotropis* species, which is was in accordance with previous works using morphology and DNA markers (Wang et al. 2017, 2019; Zhang et al. 2019). Further studies on representatives of this family are needed and will be useful for species identification and conservation.

**Figure 1.**
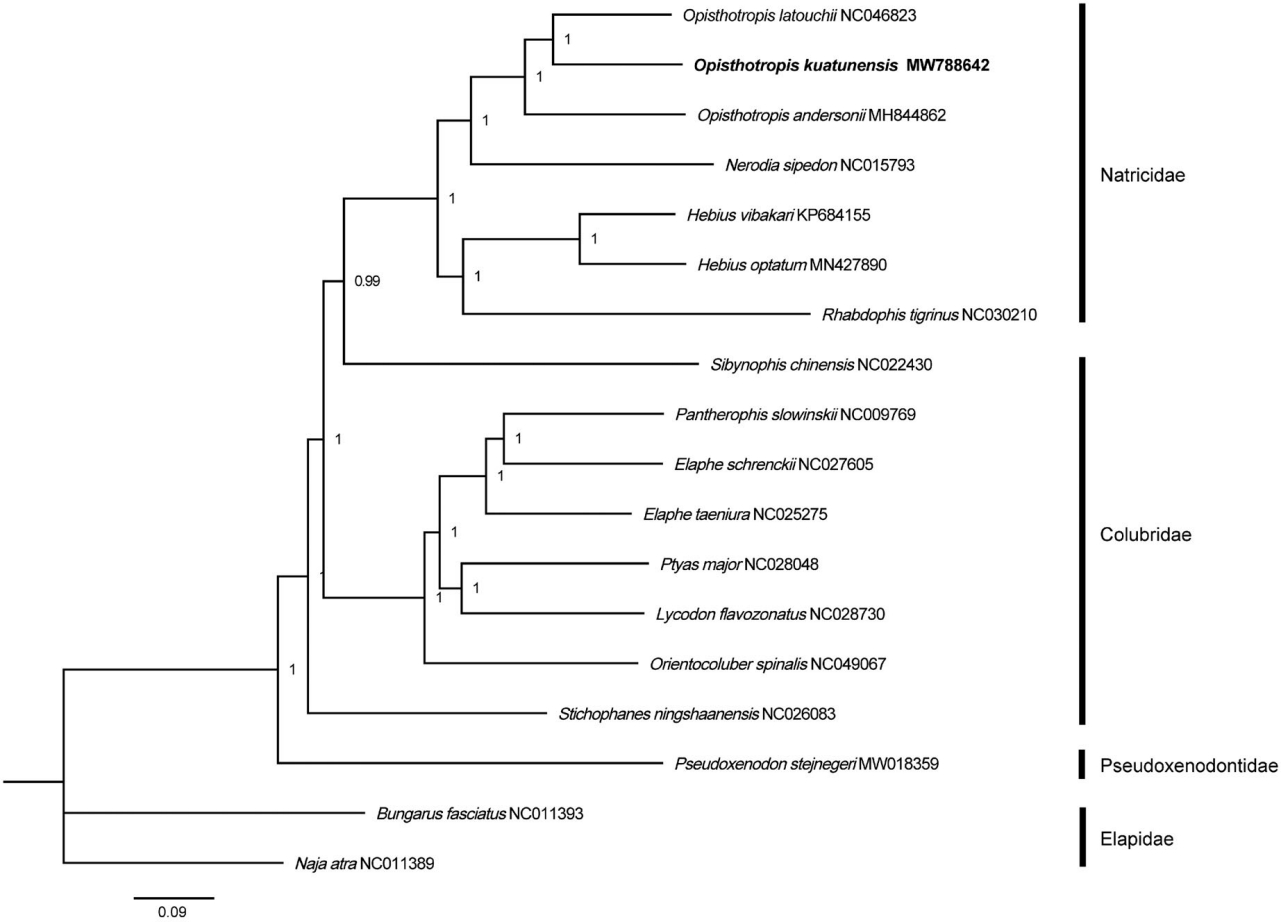
Bayesian tree based on the complete mitochondrial genome. Numbers at the nodes represent Bayesian posterior probabilities. The sample sequenced in this study is highlighted in bold.

## Data Availability

The mitogenome data supporting this study have been deposited in GenBank under the accession number MW788642 and is also openly available at https://www.ncbi.nlm.nih.gov/nuccore/MW788642.1. The associated BioProject, SRA, and Bio-Sample numbers are PRJNA636742, SRR13516389, and SAMN17521695, respectively.
